# *Mycobacterium tuberculosis* Rv1096 protein: gene cloning, protein expression, and peptidoglycan deacetylase activity

**DOI:** 10.1186/1471-2180-14-174

**Published:** 2014-06-30

**Authors:** Shufeng Yang, Fei Zhang, Jian Kang, Wenli Zhang, Guoying Deng, Yi Xin, Yufang Ma

**Affiliations:** 1 Department of Biochemistry and Molecular Biology, Dalian Medical University, 9 W Lushun South Road, Dalian 116044, China; 2 Department of Microbiology, Dalian Medical University, Dalian 116044, China; 3 Department of Biotechnology, Dalian Medical University, Dalian 116044, China; 4 Liaoning Provincial Core Lab of Glycobiology and Glycoengineering, Dalian 116044, China

**Keywords:** *Mycobacterium tuberculosis*, Cell wall, Rv1096, Peptidoglycan deacetylase, Lysozyme

## Abstract

**Background:**

Many bacteria modulate and evade the immune defenses of their hosts through peptidoglycan (PG) deacetylation. The PG deacetylases from *Streptococcus pneumonia, Listeria monocytogenes* and *Lactococcus lactis* have been characterized. However, thus far, the PG deacetylase of *Mycobacterium tuberculosis* has not been identified.

**Results:**

In this study, we cloned the *Rv1096* gene from the *M. tuberculosis* H37Rv strain and expressed Rv1096 protein in both *Escherichia coli* and *M. smegmatis.* The results showed that the purified Rv1096 protein possessed metallo-dependent PG deacetylase activity, which increased in the presence of Co^2+^. The kinetic parameters of the PG deacetylase towards *M. smegmatis* PG as a substrate were as follows: K_m_, 0.910 ± 0.007 mM; V_max,_ 0.514 ± 0.038 μMmin^-1^; and K_cat_ = 0.099 ± 0.007 (S^-1^). Additionally, the viability of *M. smegmatis* in the presence of over-expressed Rv1096 protein was 10^9^-fold higher than that of wild-type *M. smegmatis* after lysozyme treatment. Additionally, light microscopy and scanning electron microscopy showed that in the presence of over-expressed Rv1096 protein, *M. smegmatis* kept its regular shape, with an undamaged cell wall and smooth surface. These results indicate that Rv1096 caused deacetylation of cell wall PG, leading to lysozyme resistance in *M. smegmatis*.

**Conclusion:**

We have determined that *M. tuberculosis* Rv1096 is a PG deacetylase. The PG deacetylase activity of Rv1096 contributed to lysozyme resistance in *M. smegmatis*. Our findings suggest that deacetylation of cell wall PG may be involved in evasion of host immune defenses by *M. tuberculosis*.

## Background

*Mycobacterium tuberculosis*, the agent of tuberculosis, is associated with greater morbidity and longer dormancy infection times in humans than any other type of bacterial illness. Approximately one third of the population worldwide are infected with *M. tuberculosis*, which causes nearly two million deaths each year [1]. The chronic state and dormancy of tuberculosis implies that *M. tuberculosis* has developed sophisticated strategies to modify and evade the innate and adaptive immune surveillance mechanisms of humans [2].

*M. tuberculosis* has a distinctive cell wall structure, which is called the “core” mycolyl arabinogalactan-peptidoglycan (mAGP) complex. It is well-known that the bacterial cell wall is a reservoir for many essential biomolecules that interact with the surrounding environment. Peptidoglycan (PG) the skeletal structure of the cell wall, enables bacteria to resist osmotic pressure. The nucleotide-binding oligomerization domain (Nods) proteins in host cells, which have been identified as unique intracellular pattern-recognition receptors of PG and PG-derived muropeptides, are potential virulence factors [3,4]. Therefore, bacteria may have developed PG modification properties to modulate Nods-mediated host surveillance [3]. This is evidenced from the role PG plays in the pathogenesis of *Streptococcus pneumoniae*[5], *Listeria monocytogenes*[6] and *Helicobacter pylori*[7].

Deacetylation of PG in several bacterial species, such as *S. pneumonia*, *L. monocytogenes* and *Lactococcus lactis*, prevents fusion of the phagosome with macrophage lysozyme [5,8-13]. Although peptidoglycan deacetylase has been identified in some bacteria [5-8], it has not yet been identified in *M. tuberculosis*.

*M. smegmatis* is commonly used as a model for studying gene function in *M. tuberculosis* because it proliferates rapidly and is non-pathogenic. *M. smegmatis* and *M. tuberculosis* have the same basic cell wall structure [14]. Therefore, *M. smegmatis* peptidoglycan can be used as a substrate to investigate peptidoglycan deacetylase activity.

In this study, we cloned *M. tuberculosis Rv1096* and expressed Rv1096 protein in *Escherichia coli* and *M. smegmatis.* We determined the peptidoglycan deacetylase activity of purified Rv1096 and its biochemical characteristics. We also investigated whether the Rv1096 protein in *M. smegmatis* was lysozyme resistant.

## Methods

### Bacterial strains and growth conditions

*E. coli* NovaBlue (Novagen, Madison, WI) and ER2566 (Novagen) strains were routinely grown in Luria-Bertani media (LB, Invitrogen, Carlsbad, CA). The *M. smegmatis* mc^2^155 (ATCC, USA) strain was grown in LB broth containing 0.05% (v/v) Tween 80 (LBT) or LB agar at 37°C. Antibiotics were added at appropriate concentrations if needed. To prepare PG, *M. smegmatis* mc^2^155 was grown in M9 minimal glucose medium (12.8 g sodium phosphate heptahydrate, 3 g potassium phosphate monobasic, 0.5 g sodium chloride, 1 g ammonium chloride, 0.24 g magnesium sulfate, 4 g glucose and 11.1 mg calcium chloride per L).

### *Rv1096* cloning and expression vector construction

The *Rv1096* was amplified from *M. tuberculosis* H37Rv genomic DNA (Colorado State University, USA) using Pfu DNA polymerase with *Rv1096* primer 1 (5′ TT*CATATG*CCGAAGCGACCCGACAAC 3′; the *Nde*I site is italics) and *Rv1096* primer 2 (5′ GGC*AAGCTT*TACGCACCGTTATTTGGC 3′; the *Hin*dIII site is italics). The 876 bp PCR product was ligated to a pJET1.2 blunt vector to generate a pJET-*Rv1096* plasmid, the presence of which was confirmed by DNA sequencing. *Rv1096* was ligated to the *Nde*I and *Hin*dIII sites of pColdII (Takara, Dalian, China) to generate the pColdII-*Rv1096* plasmid for expression in *E. coli. Rv1096* was also ligated to the *Nde*I and *Hin*dIII sites of pVV2 (Colorado State University, USA) to obtain the pVV2-*Rv1096M. smegmatis* expression plasmid (Table 1).

**Table 1:** Bacteria and plasmids

**Bacteria and plasmids**	**Relevant characteristic(s)**	**Resource**
**Strains**		
*E. coli* NovaBlue	Used for cloning and propagation of plasmids	Novagen
*E. coli* ER2566	Used for expression of Rv1096 protein	Novagen
*M. smegmatis*	mc^2^155 strain, used for expression of Rv1096 protein and preparation of peptidoglycan	ATCC
*E. coli* ER2566/*Rv1096*	*E. coli* ER2566 carrying pColdII-*Rv1096* plasmid	This work
*M. smegmatis*/*Rv1096*	*M. smegmatis* mc^2^155 carrying pVV2-*Rv1096* plasmid	This work
**Plasmids**		
pJET1.2/blunt vector	Carries *amp*^*R*^ gene; used for cloning PCR product	Fermentas
pColdII-*Rv1096*	Carries *amp*^*R*^ gene; used for expression Rv1096 protein in *E. coli* ER2566	This work
pVV2-*Rv1096*	Carries *kan*^*R*^ gene; used for expression of Rv1096 protein in *M. smegmatis* mc^2^155	This work

### Expression and purification of Rv1096 protein

The pColdII-*Rv1096* plasmid was transformed into *E. coli* ER2566 cells (Novagen) by a chemical transformation method [15]. *E. coli* ER2566 harboring the pColdII-*Rv1096* plasmid (ER2566/*Rv1096*, Table 1) was grown in 300 ml of LB broth containing ampicillin (100 μg/ml) at 37°C. Isopropyl-D-thiogalactopyranoside at a final concentration of 1 mM was added to the culture when the OD_600_ reached 0.5, after which the culture was incubated at 16°C for 24 h.

The pVV2-*Rv1096* plasmid was transformed into *M.* smegmatis mc^2^155 using an electroporation method [15]. *M. smegmatis* mc^2^155 harboring the pVV2-*Rv1096* plasmid (*M. smegmatis*/*Rv1096*, Table 1) was grown in 300 ml of LBT broth with kanamycin at 50 μg/ml at 37°C for 24 h.

The cultures were centrifuged at 5000 × g for 15 min and the cell pellets were resuspended in 5 ml of lysis buffer (500 mM Tris-HCl, pH 8.0, 20 mM NaCl and 20% glycerol) with 1 mM phenylmethyl sulfonyl fluoride. After sonication, the lysates were centrifuged at 15000 × g for 20 min and the supernatant fraction was loaded onto a Ni-NTA column (Qiagen, Hilden, Germany) by gravity flow. The column was washed with 20 ml of wash buffer (20 mM Tris-HCl, pH 8.0, 500 mM NaCl, 20% glycerol and 30 mM imidazole). The purified protein was eluted with 10 ml of elution buffer (20 mM Tris-HCl, pH 8.0, 500 mM NaCl and 200 mM imidazole), and the first 3 ml was collected for sodium dodecyl sulfate polyacrylamide gel electrophoresis (SDS-PAGE) and western blotting, as well as deacetylase activity detection. The purified protein (1.25 μg) was subjected to 12% SDS-PAGE and then transferred to a nitrocellulose membrane (PALL, NY, USA) in blotting buffer (20 mM Tris-base, 150 mM glycine and 20% methanol, pH 8.3). After blocking with 10% non-fat dry milk in TBST buffer (10 mM Tris-HCl, pH 8.0, 150 mM NaCl and 0.05% Tween 20), the membrane was incubated with a monoclonal (anti)-polyhistidine His-1 antibody (1:5000; Sigma-Aldrich). The membrane was washed with TBST buffer three times and then incubated with alkaline-phosphatase conjugated anti-mouse-IgG (1:2500, Sigma-Aldrich). The His_6_-tagged-protein band was visualized with 5-bromo-4-chloro-3-indolyl phosphate and nitro blue tetrazolium (Sigma-Aldrich) solution.

### Preparation of *M. smegmatis* PG

*M. smegmatis* PG was prepared from cell wall fractions as described previously [16-18]. Briefly, a 500 ml culture of *M. smegmatis* mc^2^155 in M9 minimal glucose medium was harvested when the OD_600_ reached 0.6, after which the cells were washed three times with pre-cooled phosphate buffered saline (PBS: 137 mM NaCl, 2.7 mM KCl, 10 mM Na_2_HPO_4_, 2 mM KH_2_PO_4_, pH 7.0). The pellets were resuspended in distilled water to 0.2 g/ml, mixed with an equal volume of boiling 8% SDS added drop-wise with continuous boiling for 30 min. A cell-wall-enriched fraction was obtained by centrifugation at 100,000 × g at 20°C for 60 min, followed by three washes with pre-cooled PBS. The pellet was washed with distilled water at least six times to remove the SDS. The sample was resuspended in 5 ml of buffer (10 mM Tris-HCl and 10 mM NaCl, pH 7.0) and then sonicated for 5 min. α-amylase and imidazole were added to the sample at final concentrations of 100 μg/ml and 0.32 M, respectively, and the solution was incubated at 37°C for 2 h to remove glycogen. Afterwards, proteinase K was added to the sample at a final concentration of 100 μg/ml, followed by incubation at 37°C for 1.5 h to remove lipoprotein. The proteinase K solution was then inactivated by addition of an equal volume of boiling 8% SDS with vigorous stirring for 15 min. The mixture was ultracentrifuged at 100,000 × g at 20°C for 30 min. The pelleted material was washed as described above. The resulting mAGP (mycolyl-arabinogalactan-peptidoglycan) complex was washed with acetone and dried under a vacuum. Mycolic acids were removed with 1% potassium hydroxide in methanol at 37°C for 72 h. After room temperature centrifugation at 27,000 × g for 30 min, the pelleted arabinogalactan-PG was washed with distilled water twice and dried under a vacuum. Arabinogalactan was removed by washing with 49% hydrofluoridic acid at 4°C for 120 h with stirring. The resulting PG was pelleted by room temperature centrifugation at 27,000 × g for 30 min and then washed as described above. The PG was dissolved in 50 mM HEPS buffer (pH 7.0) at 1 mg/ml until further use.

### Deacetylase activity assays

The acetyl group released from the PG was measured using an acetic acid detection kit (Roche, Darmstadt, Germany). Briefly, Rv1096 protein (2.88 μg/ml) prepared from ER2566/*Rv1096* and *M. smegmatis/Rv1096* were separately incubated with *M. smegmatis* PG. The reactions were performed at 37°C for 30 min and stopped by 10 min boiling. After room temperature centrifugation at 5000 × g for 10 min, the supernatant was collected for acetic acid measurement using a spectrophotometric assay accordingly to the kit instructions.

The kinetic properties of the Rv1096 protein toward *M. smegmatis* PG were determined as described previously [19]. The molarity of *M. smegmatis* PG was calculated based the assumption that *M. smegmatis* PG is primarily composed of repeat units of GlcNAc-MurNAc (MurNGlyc)-L-Ala-D-Glu-A_2_pm, MW 868.8 [20-22]. First, the initial velocity was evaluated according to the duration of each reaction (5, 10, 15, 30 or 45 min) and the Rv1096 concentration (1.22, 2.88 or 3.65 μg/ml) curves. Then, the optimal conditions for the enzymatic reactions were determined. Based on the initial velocity and the optimal conditions that we identified, the steady-state kinetic parameters were determined by a Lineweaver-Burke plot.

### Lysozyme susceptibility assays

To investigate whether the Rv10196 protein contributed to lysozyme resistance in *M. smegmatis*, wild-type *M. smegmatis* or *M. smegmatis/Rv1096* with over-expressed Rv1096 protein were treated with lysozyme. Both bacterial strains were incubated in LBT medium at 37°C. When the OD_600_ reached ~0.2, the cultures were divided into two equal volumes parts. One part was treated with lysozyme (Sigma-Aldrich) at a final concentration of 200 μg/ml; the other was not given this treatment. Bacterial growth was monitored by measuring the optical density at 600 nm. Bacterial viability was evaluated by counting the number of colony forming units (CFU) per milliliter on LB agar [23].

### Morphology of the *M. smegmatis* strains after lysozyme treatment

Light microscopy and electron microscopy were used to investigate whether the Rv1096 protein affected the morphology of *M. smegmatis* in the presence of lysozyme. Bacteria that were treated with lysozyme for 9 h were harvested by centrifugation at 4,500 × g at 4°C for 10 min, after which the pellets were washed with sterilized 1 M PBS (pH 7.0), three times. Samples were prepared for Ziehl-Neelsen acid-fast staining as described previously [24], and observed under a light microscope (Olympus CHB, Japan). The cells for electron microscopic analysis were fixed with 2.5% glutaraldehyde, followed by post-fixation at room temperature for 2 h with 1% osmium tetroxide. The samples were dehydrated with ethanol, which was replaced with liquid carbon dioxide by critical point drying. The dried samples were applied to a silicon wafer slide and sputter-coated with gold before examination by an electronic microscope (JSM-6360 scanning electron, JEOL, Japan).

### Statistical analysis

Data are summarized as mean value ± standard deviation (SD). Data were assessed by two-tailed unpaired *t* tests. A p value of <0.05 was considered statistically significant.

## Results

### Rv1096 shares homology with other deacetylases

**Figure 1:**
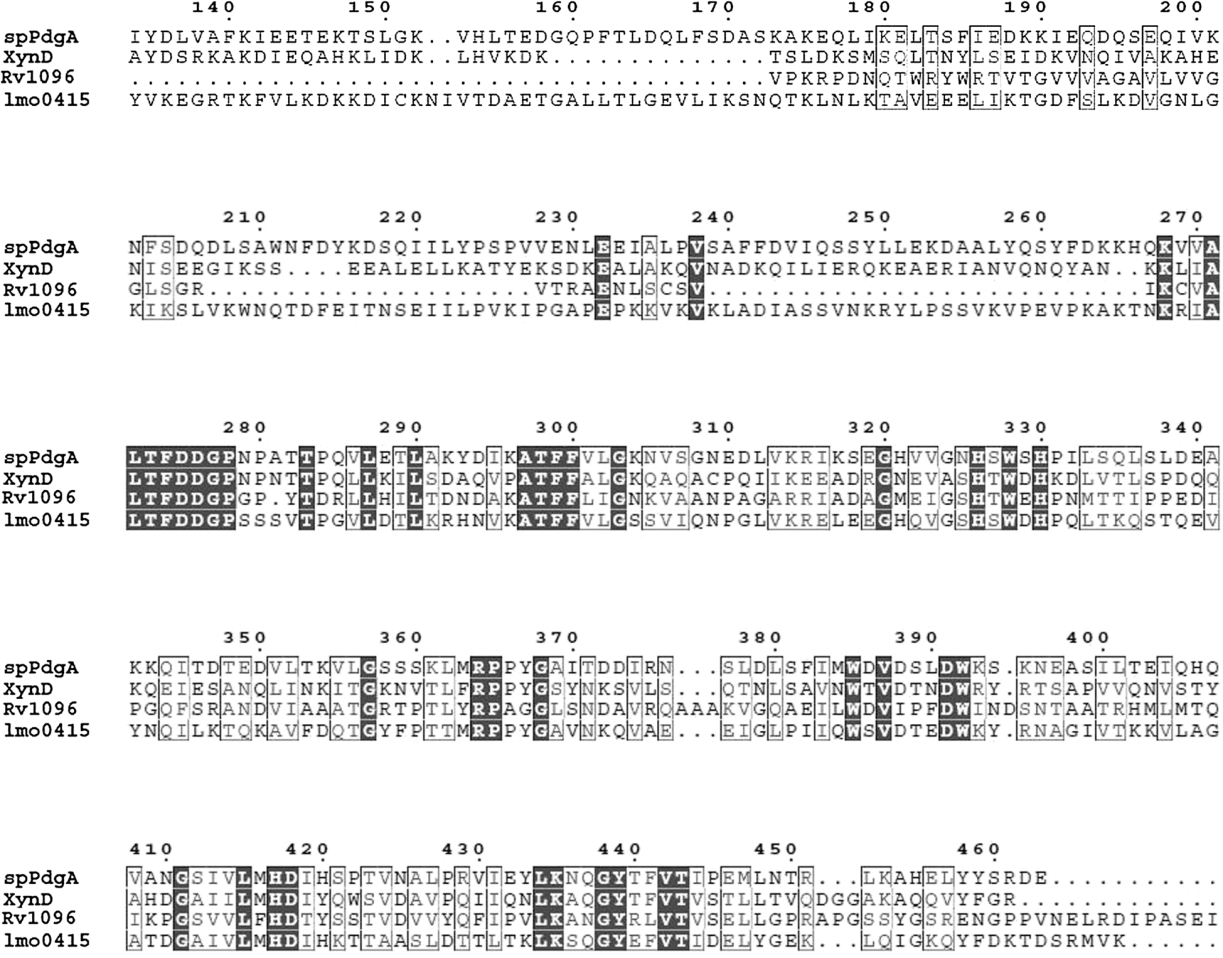
**Multiple sequence alignment of Rv1096, *sp *PgdA, lmo0415 and XynD proteins.** spPgdA, *S. pneumoniae* peptidoglycan GlcNAc deacetylase (gi:14972969); lmo0415, *L. monocytogenes* peptidoglycan GlcNAc deacetylase (gi:16409792); XynD, *L. Lactis* peptidoglycan GlcNAc deacetylase (gi:281490824). Black regions indicate identical residues in the four proteins, while residues conserved between at least two of the proteins are marked by boxes. Two catalytic histidine residues (H-326 and H-330) are conserved among Rv1096 and the other three deacetylases [10]. Rv1096 contains the metal ligand sites, Asp (D-275), Arg (A-295), Asp (D-391) and His (H-417) residues, which were identified in the *S. pneumonia* PgdA protein.

The amino acid sequences of the Rv1096 protein and other known polysaccharide deacetylases [5,8-12] were compared by Multalin analysis. The *S. pneumoniae* PgdA protein (*sp*PdgA), *L. monocytogenes* PgdA (lmo0415) and *L. lactis* PgdA (XynD) were identified as N-acetylglucosamine deacetylase proteins containing CE-4 NodB domains [10,25,26]. Rv1096 also contained a CE-4 NodB domain. Rv1096 shared 31.6% sequence identity with the *S. pneumoniae* PgdA protein, whose deacetylase domain has recently been defined as a crystal structure [10,25]. The catalytic core of the amino acids involved in deacetylase activity is highly conserved between Rv1096 and *S. pneumoniae* PgdA proteins (Figure 1).

### Rv1096 overexpressed in *E. coli* and *M. smegmatis* is a soluble protein

**Figure 2:**
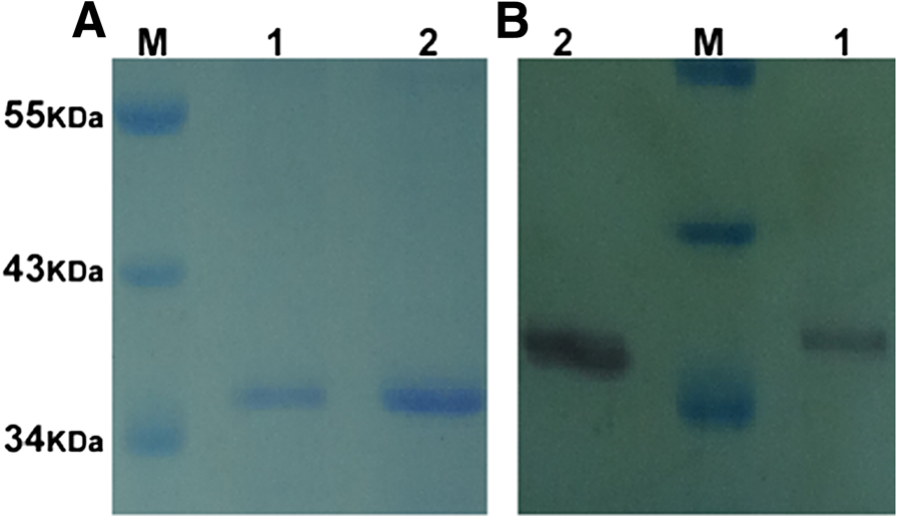
**Rv1096 protein analysis.** SDS-PAGE **(A)** and western blot **(B)** analysis of purified Rv1096 protein. *Lane* 1, purified Rv1096 protein over-expressed in *M. smegmatis*; *Lane* 2, purified Rv1096 protein over-expressed in *E. coli.* M, PageRuler™ Prestained Protein Ladder (MBI Fermentas, Lithuania).

Soluble Rv1096 protein, over-expressed in both *E. coli* and *M. smegmatis,* was purified by Ni-NTA affinity chromatography. The purified Rv196 protein was analyzed by SDS-PAGE and western blotting (Figure 2). The results showed that purified Rv1096 had a molecular weight of 35 kDa.

### Rv1096 exhibits peptidoglycan deacetylase activity

**Figure 3:**
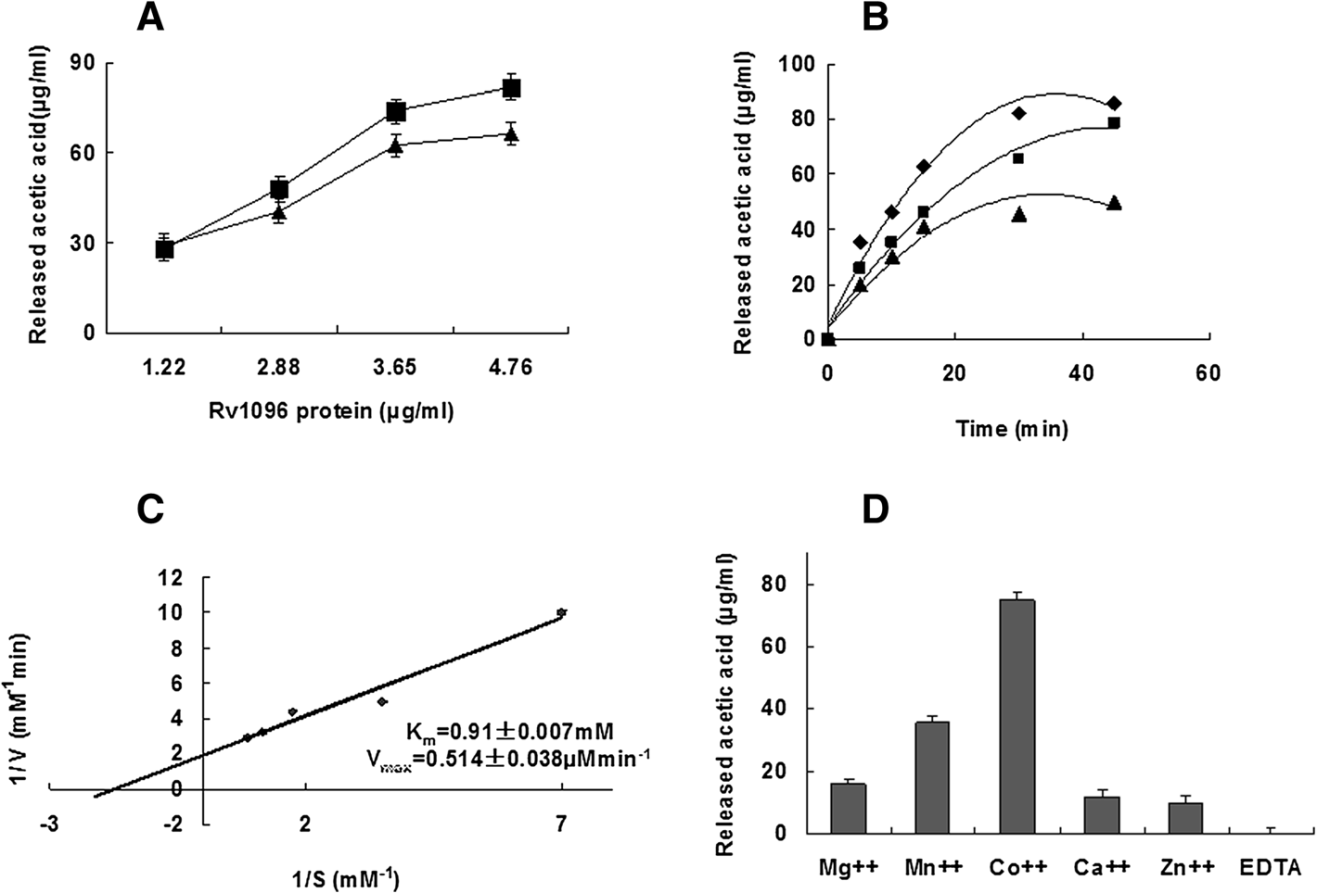
**PG deacetylase activity of purified Rv1096 protein. A)** Acetic acid released by the Rv1096 protein over-expressed in *E. coli* and *M. smegmatis*. PG (1 mg/ml) from wild-type *M. smegmatis* was used as a substrate and mixed with different concentrations of purified Rv1096 (1.22, 2.88, 3.65 or 4.74 μg/ml)*.* After incubation at 37°C for 30 min, acetyl group release was detected using an acetic acid kit. Squares indicate Rv1096 protein over-expressed in *E. coli* and triangles indicate Rv1096 protein over-expressed in *M. smegmatis*. Values are means ± SD. **B)** Time course and concentration curve for Rv1096. Purified Rv1096 protein at 1.22, 2.88 or 3.65 μg/ml was incubated with *M. smegmatis* PG (1 mg/ml) substrate at 37°C for 5, 10, 15, 30 and 50 min. Plotted values are means ± SD. **C)** K_m_ and V_max_ values for Rv1096 PG deacetylase activity. Kinetic parameters were calculated by a double reciprocal plot. **D)** Rv1096 protein exhibited a metallo dependent enzymatic activity. Various divalent cations (Mg^2+^, Mn^2+^, Co^2+^, Ca^2+^or Zn^2+^) were added to a final concentration of 0.5 μM. Values are mean ± SD.

To assess its deacetylase activity, Rv1096 protein at 1.22, 2.88, 3.65 or 4.74 μg/ml was incubated with *M. smegmatis* PG at 1 mg/ml. The acetyl group released from PG was measured using an acetic acid detection kit (Roche Diagnostics, Germany). The results revealed that the purified Rv1096 protein over-expressed in both *E. coli* and *M. smegmatis* exhibited peptidoglycan deacetylase activity (Figure 3A). There was no significant difference between the Rv1096 proteins prepared from either bacterium in terms of their specific enzymatic activities (p > 0.05). Therefore, the Rv1096 protein prepared from *E. coli* was used for the following enzyme kinetics experiments as it was easier to prepare and produced a greater yield than that produced in *M. smegmatis*.

According to the time versus concentration curve (Figure 3B), when the Rv1096 protein concentration was 2.88 μg/ml, acetic acid was released at a constant rate over a 30 min period. Therefore, the initial velocity range fell within 30 min, and the optimal concentration for Rv1096 was 2.88 μg/ml. The optimal deacetylation reaction conditions were determined by changing the pH and temperature of the reaction. From this, the optimal pH was found to be 7.0 and the optimal temperature 37°C (data not shown). The kinetic parameters were calculated by a double reciprocal plot (Figure 3C): K_m_ = 0.910 ± 0.007 mM; V_max_ = 0.514 ± 0.038 μM min^-1^; and K_cat_ = 0.099 ± 0.007 (S^-1^).

As shown in Figure 1, Rv1096 contained the same Asp-His-His conserved residues known to interact with Co^2+^ in *S. pneumoniae* PgdA. To ensure that Rv1096 was also a metallo-dependent deacetylase, various divalent cations (Mg^2+^, Mn^2+^, Co^2+^, Ca^2+^ or Zn^2+^) were added to the reaction buffer, each at a final concentration of 0.5 μM; EDTA at 50 μM served as a control. The results showed that the enzymatic reactivity reached the highest level in the presence of Co^2+^; however, enzymatic activity was lost in the presence of EDTA (Figure 3D). Therefore, we determined that Rv1096 is a metallo-dependent PG deacetylase.

### *M. smegmatis/Rv1096* exhibits lysozyme resistance

**Figure 4:**
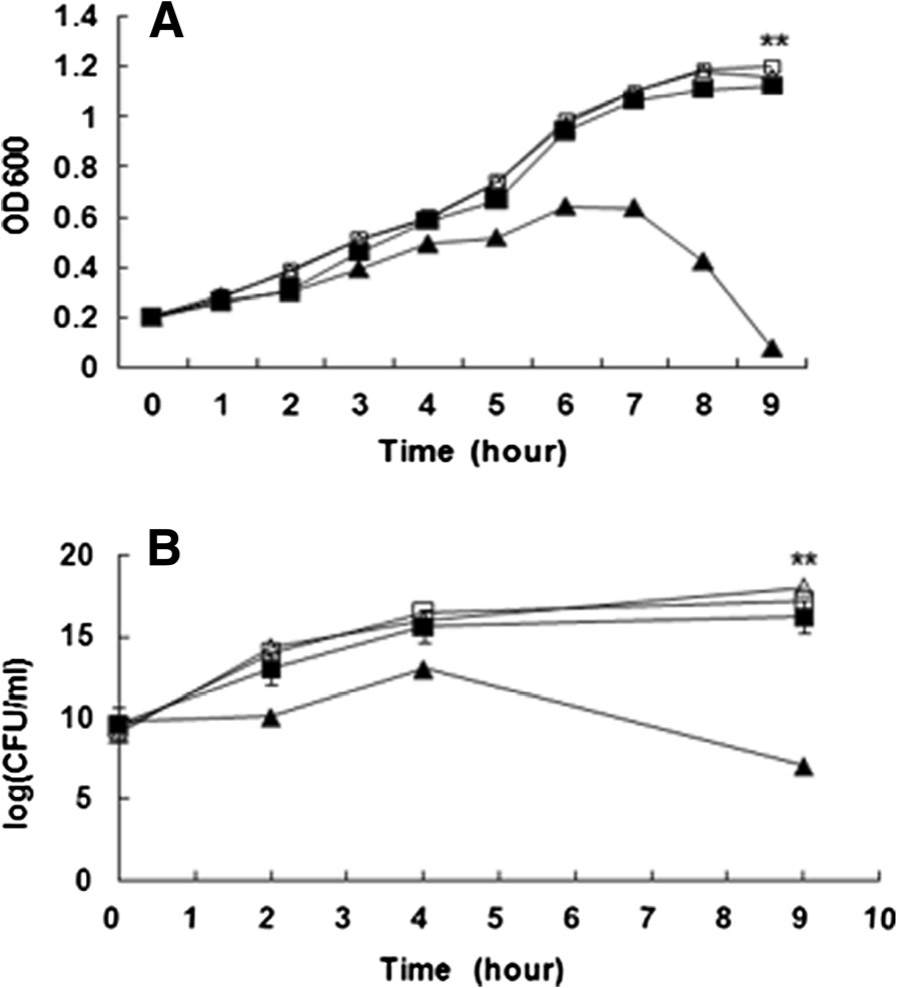
**Lysozyme susceptibility assay. A)** Lysozyme treatment growth curves for *M. smegmatis/Rv1096* and wild-type *M. smegmatis. M. smegmatis/Rv1096* (square) and wild-type *M. smegmatis* (triangle) were grown in LBT medium at 37°C to an OD_600_ of 0.2; the cultures were then divided into two parts. One part (closed symbol) was treated with lysozyme, the other part was not. Three microliter samples from each culture were collected at 1 h intervals for OD_600_ measurements. *M. smegmatis/Rv1096* showed significantly greater resistance to lysozyme than did wild-type *M. smegmatis* (**P < 0.01). Values are means ± SD. **B)** Cell survival curves for *M. smegmatis/Rv1096* and wild-type *M. smegmatis* under lysozyme treatment. *M. smegmatis/Rv1096* (square) and wild-type *M. smegmatis* (triangle) were each grown in LBT medium at 37°C to an OD_600_ of 0.2, then the cultures were divided into two parts. One part (closed symbol) was treated with lysozyme, the other part was not. Three microliter culture samples were collected at 1 h intervals to measure CFU/ml. *M. smegmatis/Rv1096* exhibited greater cell survival than that of the wild-type bacterium (**P < 0.01). Values are means ± SD.

To determine the contribution of Rv10196 protein to *M. smegmatis* resistance to lysozyme, *M. smegmatis/Rv1096* and wild-type *M. smegmatis* cultures were divided into two parts at the beginning of the exponential growth phase. Test samples received 200 μg/ml lysozyme, unlike the control samples. As shown in Figure 4A, the wild-type *M. smegmatis* culture suspension treated with lysozyme lost its opaque, hazy appearance, becoming transparent at the end of the exponential growth phase, or shortly after reaching stationery phase. Its OD_600_ and CFU values decreased, indicating that cell lysis took place in the wild-type lysozyme*-*treated *M. smegmatis*. The *M. smegmatis/Rv1096* growth curves for lysozyme treatment showed almost no difference to the lysozyme-untreated group, suggesting that Rv10196 protein contributed to *M. smegmatis* resistance to lysozyme degradation. There was also no significant difference between the *M. smegmatis/Rv1096* and wild-type *M. smegmatis* treated with lysozyme in terms of bacterial viability within the initial four hours of growth (Figure 4B). However, viable wild-type *M. smegmatis* bacteria decreased rapidly after lysozyme treatment for 4 h. A significant difference (P < 0.01) in viability was observed between *M. smegmatis/Rv1096* and wild-type *M. smegmatis* after lysozyme treatment for 9 h. About 10^7^ wild-type *M. smegmatis* cells survived, whereas only 10^16^*M. smegmatis*/*Rv1096* cells survived.

### The *M. smegmatis/Rv1096* cell wall was undamaged by 9 h of lysozyme treatment

**Figure 5:**
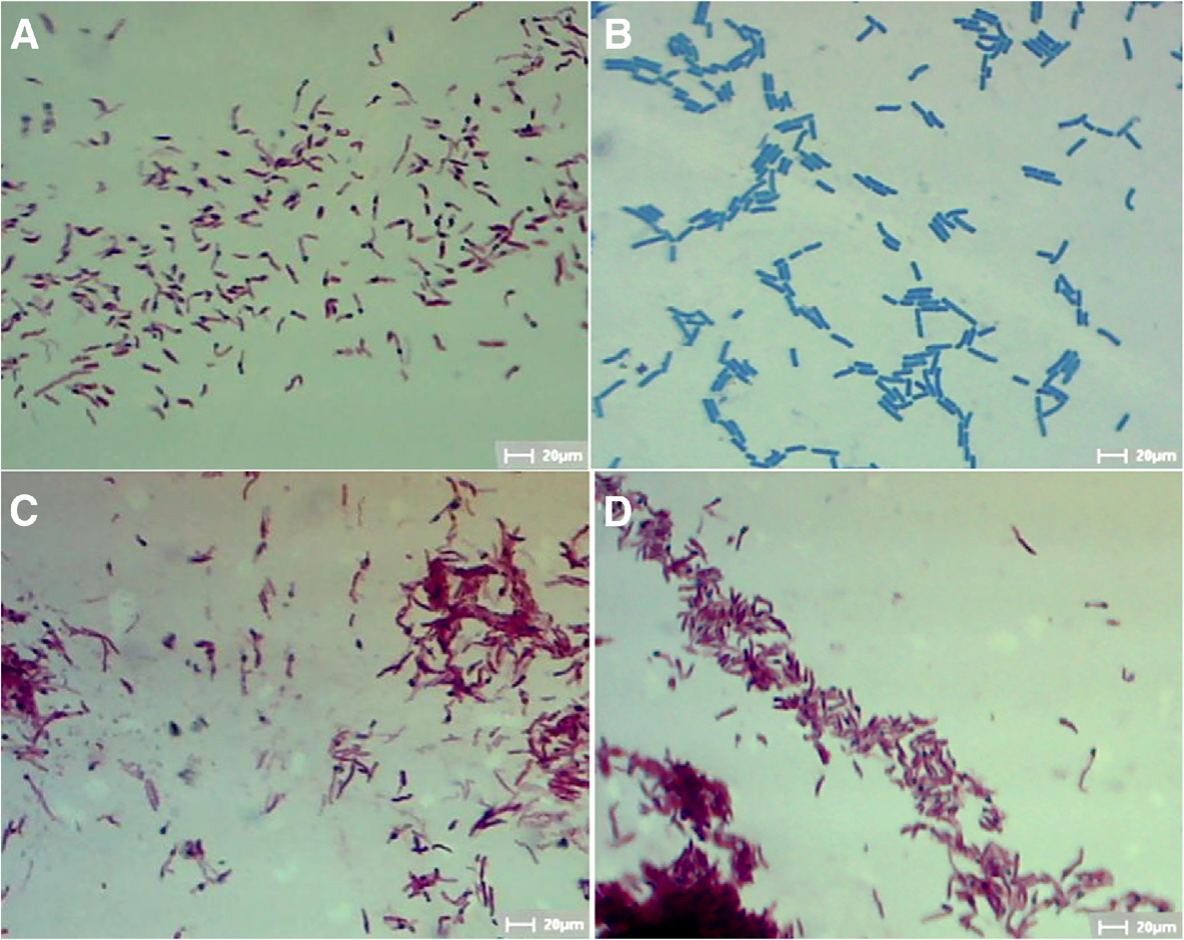
**Acid-fast staining of *M. smegmatis/Rv1096 *and wild-type cells. A)** Wild-type *M. smegmatis* without lysozyme treatment, **B)** wild-type *M. smegmatis* with lysozyme treatment, **C)***M. smegmatis/Rv1096* without lysozyme treatment and, **D)***M. smegmatis/Rv1096* with lysozyme treatment (×1000). Lysozyme treatment was for 9 h.

**Figure 6:**
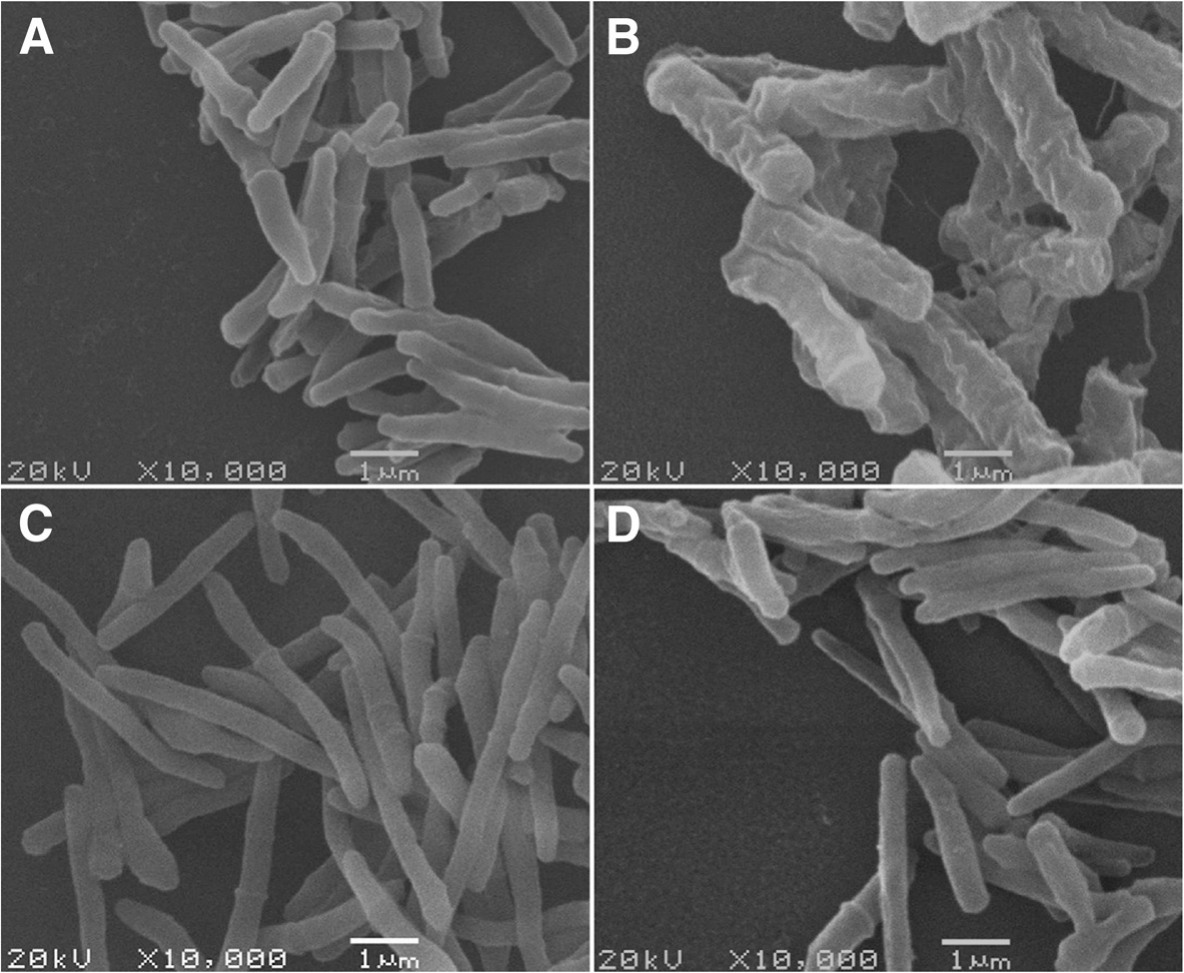
**Scanning electron micrographs of *M. smegmatis/Rv1096 *and wild-type *M. smegmatis*. A)** Wild-type *M. smegmatis* without lysozyme treatment, **B)** wild-type *M. smegmatis* with lysozyme treatment, **C)***M. smegmatis/Rv1096* without lysozyme treatment and, **D)***M. smegmatis/Rv1096* with lysozyme treatment. Lysozyme treatment was for 9 h.

Because the most apparent differences in bacterial growth and viability were observed (Figures 4A and B) after treatment with lysozyme for 9 h, morphological observations were performed at this time point. The results of the Ziehl-Neelsen acid-fast staining showed that wild-type *M. smegmatis* lost its acid-fastness and became blue dyed, whereas *M. smegmatis/Rv1096* retained its acid-fastness (Figure 5). Scanning electronic microscopy (SEM) showed that the wild-type *M. smegmatis* had an irregular appearance (enlarged shape, destructed cell wall and wrinkled surface) in the presence of lysozyme, whereas *M. smegmatis/Rv1096* had a regular shape, undamaged cell wall and smooth surface after 9 h lysozyme treatment (Figure 6).

## Discussion

*M. tuberculosis* Rv1096 protein, *S. pneumoniae* PgdA protein (*sp*PdgA), *L. monocytogenes* PgdA (lmo0415), and *L. lactis* PgdA (XynD) are carbohydrate esterase 4 (CE-4) superfamily members. The CE-4 superfamily includes peptidoglycan GlcNAc deacetylases, rhizobial NodB chito-oligosaccharide deacetylases, chitin deacetylases, acetyl xylan esterases, and xylanases [27]. The substrates of these enzymes are polymers or basic structures that assemble PG backbone glycan strands. In this study, Rv1096 protein, over-expressed in both *E. coli* and *M. smegmatis,* was able to deacetylate *M. smegmatis* peptidoglycan. Therefore, *M. tuberculosis* Rv1096 protein is a peptidoglycan deacetylase.

As shown in Figure 1, Rv1096 and three other deacetylases share sequence conservation at two catalytic histidine residues (H-326 and H-330) [10]. The metal ligand sites, including Asp (D-275), Arg (A-295), Asp (D-391) and His (H-417) residues, which were identified in the *S. pneumonia* PgdA protein [5,10,28], are all present in the Rv1096 protein. These highly conserved sequences in Rv1096 suggest that it may have metallo-dependence. Indeed, our results show that the enzymatic activity of Rv1096 increased after supplementation with divalent cations, especially Co^2+^. Taken together, our results suggest that Rv1096 may use similar catalytic mechanisms as the *S. pneumoniae* PgdA protein to deacetylate PG.

It has been reported that PG deacetylase contributes to lysozyme resistance in some bacterial species, such as *Bacillus cereus*[29], *S. pneumonia*[10] , *L. monocytogenes*[6] and *Shigella flexneri*[28]. Generally, pdgA mutants are more sensitive to lysozyme degradation in the stationary phase. Similarly, *M. smegmatis* over-expressing Rv1096 protein showed remarkable resistance to lysozyme at the end of log phase growth. In the present study, the viability of *M. smegmatis/Rv1096* was 10^9^-fold higher than that of wild-type *M. smegmatis* after lysozyme treatment, indicating that PG deacetylation by the Rv1096 deacetylase had increased lysozyme resistance. The morphological changes observed between wild-type *M. smegmatis* and *M. smegmatis/Rv1096* provides strong evidence that Rv1096 activity helped to preserve the integrity of the cell wall during lysozyme treatment. Wild-type *M. smegmatis* lost its acid-fastness because of the increased cell wall permeability caused by lysozyme treatment. SEM observations showed that wild-type *M. smegmatis* had a wrinkled cell surface with outward spilling of its cell contents, while *M. smegmatis/Rv1096* maintained its cell wall integrity and acid fastness. Therefore, it is likely that the functionality of the Rv1096 protein of *M. smegmatis/Rv1096* contributed to its cell wall integrity.

In fact, PG N-deacetylase has been shown to be a virulence factor in several bacteria including *S. pneumonia*[5], *S. iniae*[30]*, L. monocytogenes*[12] and *H. pylori*[7]. For example, the *S. pneumoniae* pdgA mutant (with a nonfunctional pdgA gene) developed hypersensitivity to exogenous lysozyme and decreased virulence in a mouse infection model [5]. Furthermore, *L. monocytogenes* lacking pdgA (lmo0415) was susceptible to macrophage clearance [12]. In further studies, we aim to establish whether the Rv1096 protein is a virulence factor.

## Conclusion

We identified *M. tuberculosis* Rv1096 as a PG deacetylase and found that the PG deacetylase activity of this protein contributed to lysozyme resistance in *M. smegmatis*. Our findings suggest that PG deacetylation may be involved in immune evasion by *M. tuberculosis* in its host.

## Abbreviations

PG: Peptidoglycan; mAGP: Mycolyl-arabinogalactan-peptidoglycan; Nod: Nucleotide-binding oligomerization domain; IPTG: Isopropyl-D-thiogalactopyranoside; PMSF: Phenylmethyl-sulphonyl fluoride; BCIP/NBT: 5-Bromo-4-chloro-3-indolyl phosphate/Nitro blue tetrazolium; SDS: Sodium dodecyl sulfate; CFU: Colony forming unit; OsO4: Omium tetroxide; SEM: Scanning electronic microscopy; CE-4: Carbohydrate esterase 4.

## Competing interests

The authors declare that they have no competing interests.

## Authors’ contributions

SY constructed expression vectors, prepared Rv1096 protein and conducted lysozyme susceptibility assays, deacetylase activity assays, as well as prepared this manuscript. FZ purified Rv1096 protein and determined kinetic parameters of PG deacetylase. JK performed bioinformatic analyses of Rv1096 with known PG deacetylases. WZ performed bioinformatic analysis of Rv1096 and the statistical analyses. GD prepared samples for acid-fast staining and SEM. YX participated in designing experiments of the study. YM proposed this project, designed most of experiments and prepared this manuscript. All authors read and approved the final manuscript.

## Authors’ information

Shufeng Yang (M.S.) and Guoying Deng (M.S.):Department of Microbiology, Dalian Medical University Dalian 116044, China; Fei Zhang (B.S.), Jian Kang (Ph.D.), Wenli Zhang (Ph.D.) and Yufang Ma (Ph.D.): Department of Biochemistry and Molecular Biology, Dalian Medical University Dalian 116044, China. Yi Xin (Ph.D.), Department of Biotechnology, Dalian Medical University Dalian 116044, China.
